# Peroxiredoxin 6 Peroxidase and Ca^2+^-Independent Phospholipase A_2_ Activities Are Essential to Support Male-Mouse Fertility

**DOI:** 10.3390/antiox11020226

**Published:** 2022-01-25

**Authors:** Edrian Bumanlag, Eleonora Scarlata, Cristian O’Flaherty

**Affiliations:** 1 Department of Surgery (Urology Division), McGill University, Montréal, QC H4A 3J1, Canada; edrian.bumanlag@mail.mcgill.ca (E.B.); eleonora.scarlata@affiliate.mcgill.ca (E.S.); 2 The Research Institute, McGill University Health Centre, Montréal, QC H4A 3J1, Canada; 3 Department of Pharmacology and Therapeutics, McGill University, Montréal, QC H3G 1Y6, Canada; 4 Department of Anatomy and Cell Biology, McGill University, Montréal, QC H3A 0C7, Canada

**Keywords:** antioxidant enzymes, spermatozoa, epididymal maturation, sperm capacitation, male fertility

## Abstract

Human infertility is an important health problem that affects one in six couples worldwide. Half of these cases are due to male infertility. Oxidative stress is a common culprit of male infertility, promoting lipid peroxidation and the oxidation of proteins and DNA in spermatozoa, thereby impairing motility, capacitation and fertilization. Peroxiredoxin 6 (PRDX6) possesses peroxidase and Ca^2+^-independent-phospholipase-A_2_ (iPLA_2_) activities that scavenge ROS and repair oxidized sperm membranes, respectively. PRDX6 protects spermatozoa against oxidative stress. Infertile men’s spermatozoa have impaired motility, elevated lipid peroxidation levels and DNA damage due to low PRDX6 levels. A lack of PRDX6 is associated with male-mouse infertility. Here, we determined the impact of the absence of PRDX6 peroxidase or iPLA_2_ activities on male-mouse fertility. Two-month-old male C57Bl6/J (wild-type), *Prdx6^−/−^*, C47S and D140A knock-in (peroxidase- and iPLA_2_-deficient, respectively) male mice were challenged with an in vivo oxidative stress triggered by tert-butyl hydroperoxide (t-BHP). C47S and D140A males produced smaller litters compared to wild-type controls. The t-BHP treatment promoted a lower number of pups, high levels of lipid peroxidation, tyrosine nitration, and DNA oxidation in all mutant spermatozoa compared to wild-type controls. All mutant spermatozoa had impaired capacitation and motility. In summary, both PRDX6 peroxidase and iPLA_2_ activities are essential to support male-mouse fertility.

## 1. Introduction

Currently, one in six couples are infertile, and half of these cases are caused by male infertility factors [1,2]. Various pathological conditions, notably varicocele, cancer and chemotherapy, excessive exposure to drugs and pollutants, smoking, chemotherapy, and aging may lead to male infertility [3,4,5,6,7]. These factors promote oxidative stress caused by a decrease in antioxidant enzymes and/or an increase in the levels of reactive oxygen species (ROS), such as the superoxide anion (O_2_^•−^), nitric oxide (NO^•^), hydrogen peroxide (H_2_O_2_) and peroxynitrite (ONOO^−^, the product of O_2_^•−^ and NO^•^ combination) [3,4,5,6,7,8]. While low ROS levels are necessary for the fertilization ability of the spermatozoon [9,10,11,12], high ROS levels are associated with male infertility. Oxidative stress promotes lipid peroxidation, damage of the paternal genome characterized by DNA oxidation and fragmentation in spermatozoa [13,14,15], oxidation of proteins [16,17,18], decreased energy production that impairs sperm motility [19] and decreases mitochondrial potential [20,21]. The lipid-peroxidation product 4-hydroxynonenal (4-HNE) promotes DNA mutations [22,23] and disrupts mitochondrial activity, thereby generating high levels of O_2_^•−^ that react with NO^•^ to form ONOO^−^ [24], which is responsible for protein tyrosine nitration associated with impairment of motility and capacitation [16,25,26,27].

Mammalian spermatozoa are vulnerable to oxidative stress since they have limited antioxidant protection. Indeed, peroxisomes that contain catalase (H_2_O_2_ scavenger) are removed during spermatogenesis [28,29] and their O_2_^•−^ scavenging capacity is low because SOD1 is present in low amounts due to the small amount of cytoplasm present in the ejaculated spermatozoon [30]. Their protection against H_2_O_2_ and ONOO^−^ is also limited since isoforms of glutathione peroxidases (GPXs) 1, 2, 3 and 5 are absent in human spermatozoa [31], and the mitochondrial GPX4 isoform is a structural protein of the mitochondrial sheath without antioxidant capabilities in mature spermatozoa [32]. The antioxidant protection of spermatozoa against H_2_O_2_, organic hydroperoxides and ONOO^−^ is provided by peroxiredoxins (PRDXs) [13,24,33,34,35,36]. PRDXs are Se^2+^-independent antioxidant enzymes that are conserved in all species and scavenge H_2_O_2_, organic peroxides and ONOO^−^ through their peroxidase activity [37,38,39]. PRDXs can have one (1-Cys; PRDX6) or two (2-Cys; PRDX1-5) cysteine residues within their active site [40], and they are widely localized throughout the entire sperm-cell compartments [35,36]. Among all PRDXs, only PRDX6 is found in all sperm compartments, reacts with small H_2_O_2_ concentrations required for capacitation [36,41], and possesses Ca^2+^-independent phospholipase A_2_ (iPLA_2_) and lysophosphatidylcholine acyltransferase (LPCAT), which removes oxidized phospholipids and reintegrates reduced fatty acids to repair oxidized sperm membranes, respectively [42,43].

Spermatozoa from infertile men suffering from idiopathic infertility or varicocele have elevated levels of lipid peroxidation, DNA oxidation and impaired motility associated with low PRDX6 levels [13]. *Prdx6^−/−^* male mice are subfertile, producing 50% fewer viable pups compared to age-matched wild-type controls [34]. *Prdx6^−/−^* spermatozoa have higher lipid peroxidation, DNA oxidation and fragmentation, impaired motility and lower chromatin compaction than wild-type males [34,44]. These phenotypes are aggravated by tertbutyl hydroperoxide (t-BHP), an in vivo inducer of oxidative stress [34]. The absence of PRDX6 or the inhibition of PRDX6-iPLA_2_ activity impairs sperm capacitation and leads to decreased fertilization rates, delayed pronuclei formation, and impaired sperm binding to both the zona pellucida and the oolemma during in vitro fertilization [33,34].

Since *Prdx6^−/−^* males are subfertile, displaying oxidative damage in their spermatozoa, we aimed to assess the effects of the absence of the peroxidase or iPLA_2_ activities of PRDX6 on mouse fertility using the knock-in C47S and D140A mouse strains that lacked the peroxidase or the iPLA_2_ activities of the PRDX6, respectively.

## 2. Materials and Methods

### 2.1. Chemicals and Reagents

Mouse monoclonal anti-3-nitrotyrosine and goat polyclonal anti-4-HNE primary antibodies were purchased from Abcam (Toronto, ON, Canada). Mouse monoclonal anti-8OHdG antibody was purchased from StressMarq (Victoria, BC, Canada). Goat anti-mouse (H + L) and donkey anti-goat (H + L) antibodies, both conjugated with AlexaFluor 555 were purchased from ThermoFisher Scientific (Markham, ON, Canada). Gibco Giemsa stain was purchased from FisherScientific (Toronto, ON, Canada). The other chemicals used were of at least reagent grade and purchased from Sigma-Aldrich (Milwaukee, WI, USA).

### 2.2. Animals and Treatment

We used two-month-old males C57BL/6J (wild-type), *Prdx6^−/−^*, and C47S and D140A knock-in strains, lacking PRDX6 peroxidase and iPLA_2_ activities, respectively. The generation of *Prdx6^−/−^* mice was conducted by Dr. Ye Shih Ho (Wayne State University) and Dr. Aron Fisher (University of Pennsylvania) [45]. *Prdx6^−/−^* mice were backcrossed to C57BL/6J mice to achieve > 99.9% homozygosity. C47S and D140A mice were generated using an amino-acid substitution from cysteine (C) to serine (S) and from aspartate (D) to alanine (A), respectively [46]. Mice colonies were generated and maintained at the Research Institute of McGill University Health Centre. Mice were provided food and water and were exposed to 14 h of light and 10 h of dark daily. Regulations implemented by the Canadian Council for Animal Care and approved by the Animal Care Committees of McGill University and the McGill University Health Centre were followed. Similar to Prdx6^−/−^ mice [33], C47S and D140A males have normal spermatogenesis (data not shown).

Males from each strain were intraperitoneally injected with either saline (control) or 60 µmol/100 g of body weight of t-BHP for nine consecutive days to generate control and t-BHP-treated groups, respectively [34]. Twenty-four hours post-treatment, control and treated males were placed with age-matched wild-type females (1:1 ratio) in separate cages during three consecutive matings. The presence of a vaginal plug was checked daily and considered as positive mating, and the female was separated from the male until the pups were born. The numbers of litters, pups, and unsuccessful matings (corresponding to infertile matings) were recorded. At the end of the experiment, the males were euthanized to collect spermatozoa from the cauda epididymis. Immediately after collection, both cauda epididymides were placed in 300 μL of Biggers, Whitten and Wittingham (BWW) medium composed of 91.5 mM NaCl, 4.6 mM KCl, 1.7 mM CaCl_2_, 1.2 mM KH_2_PO_4_, 1.2 mM MgSO_4_, 5.6 mM D-glucose, 0.27 mM sodium pyruvate, 44 mM sodium lactate and 20 mM HEPES (pH 7.4) for 10 min at 37 °C to let the sperm swim out. Then, fresh aliquots of spermatozoa in the BWW were used to determine sperm motility or sperm capacitation. Sperm aliquots were stored at −80 °C for further determination of 4-HNE and tyrosine-nitration levels by SDS-PAGE and immunoblotting.

### 2.3. Assessment of Sperm Motility and Cytoplasmic Droplet Retention

Sperm motility and cytoplasmic droplet retention, which are markers of epididymal maturation, were determined to have been previously studied [47]. The cauda epididymides were incubated in phosphate-buffered saline (PBS) (1 mM KH_2_PO_4_, 10 mM Na_2_HPO_4_, 137 mM NaCl, 2.7 mM KCl, pH 7.4). Cauda epididymides were placed in PBS without energetic substrates, and sperm were allowed to swim out into the PBS solution for 10 min at 37 °C. Sperm aliquots were taken at various time points (0, 30, 60, 90, 120 min) to determine total and progressive sperm motility using a computer-assisted sperm-analysis (CASA) system with Sperm Vision HR software version 1.01 (Minitube, Ingersoll, ON, Canada). The percentage of spermatozoa carrying a visible cytoplasmic droplet was recorded for both control and treated males.

### 2.4. Assessment of Sperm Capacitation

Sperm samples were incubated in BWW medium with or without capacitation inducers (5 mg/mL BSA + 25 mM HCO_3_) for 60 min at 37 °C. Samples were re-centrifuged at 600× *g* for 5 min at 20 °C. The supernatant was discarded, then samples were incubated in fresh BWW with 10 μM progesterone for 30 min at 37 °C to promote the acrosome reaction as previously described [33,34]. Samples were then fixed with 4% paraformaldehyde for 15 min at 20 °C, centrifuged at 350× *g* for 5 min at 20 °C, and the pellet was resuspended in BWW. Samples were smeared onto Superfrost slides, air dried, then incubated with 1:10 Giemsa stain diluted with distilled water for 1.5 h. After washing, slides were covered with a coverslip to determine the percentage of spermatozoa with intact and reacted acrosomes of 200 spermatozoa per sample using a Leica DM500 microscope (Opti-Tech Scientific, Montreal, QC, Canada) at 600× magnification. The percentage of acrosome reaction was defined as the percentage of Giemsa-stain-negative spermatozoa.

### 2.5. Determination of Lipid Peroxidation and Tyrosine Nitration in Non-Permeabilized Spermatozoa

We determined the levels of 4-HNE (a product of lipid peroxidation) and tyrosine nitration in sperm plasma-membrane proteins using non-permeabilized spermatozoa, as has been previously performed with modifications [16]. Non-permeabilized sperm samples were smeared onto Superfrost slides, then dried at 37 °C. Samples were rehydrated with PBS + Triton 0.1% (PBS-T) for 5 min. Samples were incubated in fresh PBS-T for 25 min. Horse serum (1%) in PBS-T and 5% goat serum in PBS-T, for anti-4-HNE and nitro-tyrosine antibodies, respectively, were used for blocking for 30 min at 37 °C. Samples were washed with PBS-T, then incubated with their respective primary antibodies overnight at 4 °C. Anti-4-HNE and anti-nitrotyrosine antibodies were diluted in 1% horse serum in PBS-T and 1% goat serum in PBS-T, respectively. Samples were washed, then incubated with 1:2000 donkey anti-goat secondary antibody diluted in PBS-T + 1% BSA or with 1:1000 goat anti-mouse secondary antibody diluted in PBS-T + 1% BSA, for anti-4-HNE and nitro-tyrosine antibodies, respectively, for 1 h at 37 °C. Samples were washed, then ProLong Antifade with DAPI was added prior to coverslip application. The negative control was incubated with secondary antibody alone. A magnification of 40× and the same exposure times were used for each sample. ImageJ was used for background fluorescence subtraction and for quantification of average relative fluorescence intensity (RFI) of at least 200 spermatozoa per sample.

### 2.6. Determination of Sperm DNA Oxidation

Sperm DNA oxidation was determined according to Ozkosem et al. (2016) with modifications [34]. Sperm samples were smeared onto Superfrost slides and air dried. Then, samples were treated with a 50 mM Tris-HCl, pH 7.4 buffer containing 1% SDS, 40 mM DTT and 1 mM EDTA for 5 min. Samples were washed and incubated with 5% goat serum in PBS-T for 1 h at 37 °C and washed. Then, samples were incubated with anti-8OHdG antibody (1:100 dilution in 1% goat serum in PBS-T) overnight at 4 °C. Samples were washed, then incubated with goat anti-mouse secondary antibody (1:2000 dilution in PBS-T supplemented with 1% BSA) for 1 h at 37 °C. Samples were rewashed and ProLong Antifade with DAPI (Molecular Probes (Eugene, OR, USA)) were added, before being covered with a coverslip. The negative control was incubated with PBS-T without the primary antibody. The specificity of the anti-8OHdG antibody was confirmed as previously described [44]. The same procedures for imaging and quantifying average RFI were done. Results of DNA oxidation were expressed as percentages of spermatozoa with a strong signal for 8OHdG.

### 2.7. Statistical Analysis

All results are presented as mean ± SEM. The normality of the data and homogeneity of variances were determined using Shapiro–Wilk and Bartlett tests, respectively. We used the two-way ANOVA and Bonferroni tests (to assess changes due to genotype and treatment) and chi-square tests as appropriate, using GraphPad Prism 5 (GraphPad Software, Inc., San Diego, CA, USA) to determine statistical differences among groups.

## 3. Results

### 3.1. The Absence of PRDX6 Peroxidase and iPLA_2_ Activities Promotes Male’s Abnormal Reproductive Outcomes

To know the impact of the absence of either PRDX6 peroxidase or iPLA_2_ activities in male fertility, we conducted controlled matings comparing the reproductive performance of C47S and D140A males with wild-type and *Prdx6^−/−^* males. During these experiments, positive matings were confirmed by the presence of a vaginal plug. We found that control wild-type males had no infertile matings, and among the treated wild-type mice, there were approximately half as many infertile matings compared to the respective mutant-strain groups (Table 1). The C47S and D140A control and treated males had similar numbers of infertile matings to the *Prdx6^−/−^* control and treated males, respectively.

**Figure 1:**
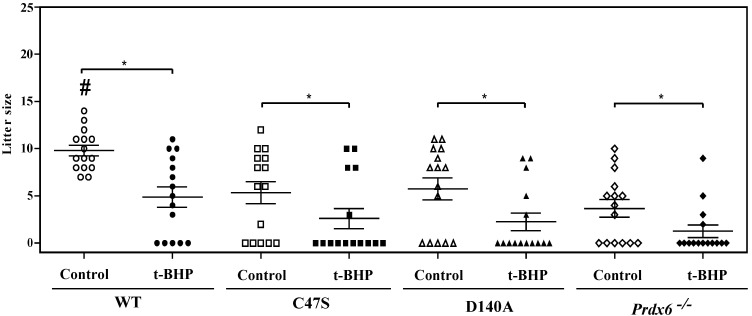
PRDX6 peroxidase or iPLA_2_ absence leads to the production of a low number of pups. Each symbol corresponds to one litter from wild-type (WT), C4, D140A or *Prdx6^−/−^* mouse strains. Open and closed symbols correspond to litters from control and t-BHP-treated males, respectively. Mean and SEM are indicated by the horizontal lines for each group. ^#^ means higher than all other groups, and * means significant differences between groups (ANOVA and Tukey tests; *p* < 0.05; *n* = 5 males for each group).

The production of pups was severely impaired by the absence of PRDX6 or its peroxidase and iPLA_2_ activities. While the control wild-type males produced the highest number of pups among all groups, mutant-strain males had significantly smaller litters than the wild-type controls (Figure 1). The t-BHP treatment lowered the pup production of the wild-type males to similar numbers to those produced by mutant male controls. Moreover, t-BHP treatment promoted the production of smaller litters in each mutant strain compared with their respective control group.

### 3.2. The Absence of PRDX6 Peroxidase and iPLA_2_ Activities Promotes Abnormal Sperm Epididymal Maturation

**Figure 2:**
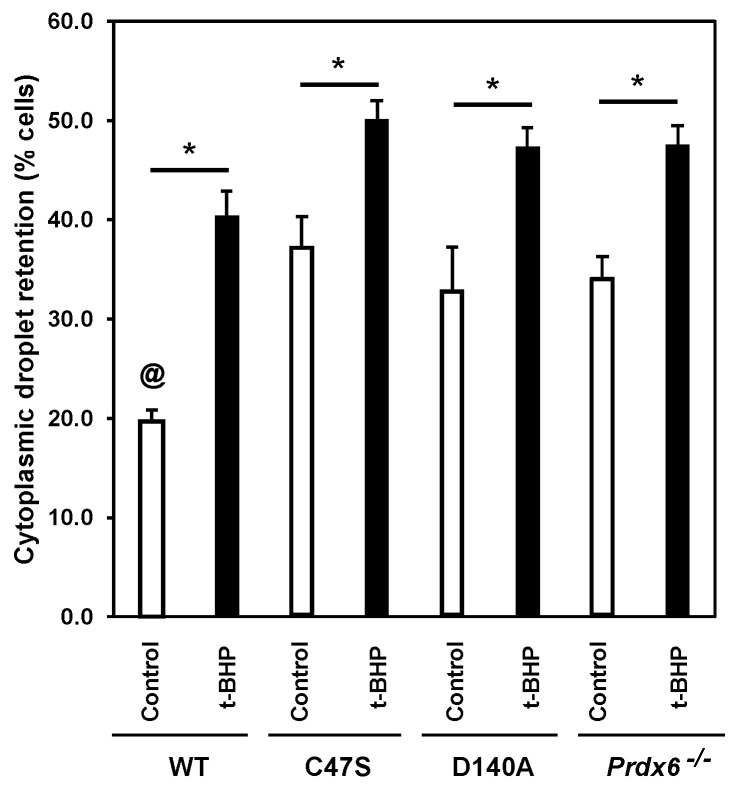
Epididymal maturation is impaired in C47S, D140A and *Prdx6^−/−^* males. ^@^ means smaller than all others. * means significant differences in the same strain. Two-way ANOVA and Bonferroni post hoc tests; *p* < 0.05 (*n* = 5 males for each group).

The cytoplasmic droplet retention (CDR) of the spermatozoon indicates abnormal epididymal maturation. The control wild-type males had the lowest percentages of spermatozoa with CDR among all the groups (Figure 2). We observed that the percentages of spermatozoa with CDR of the t-BHP-treated wild-type males were similar to those of the control mutants. The t-BHP treatment increased the percentages of spermatozoa with CDR in all the mutant strains.

### 3.3. The Absence of PRDX6 Peroxidase and iPLA_2_ Activities Increased Lipid Peroxidation in Spermatozoa

**Figure 3:**
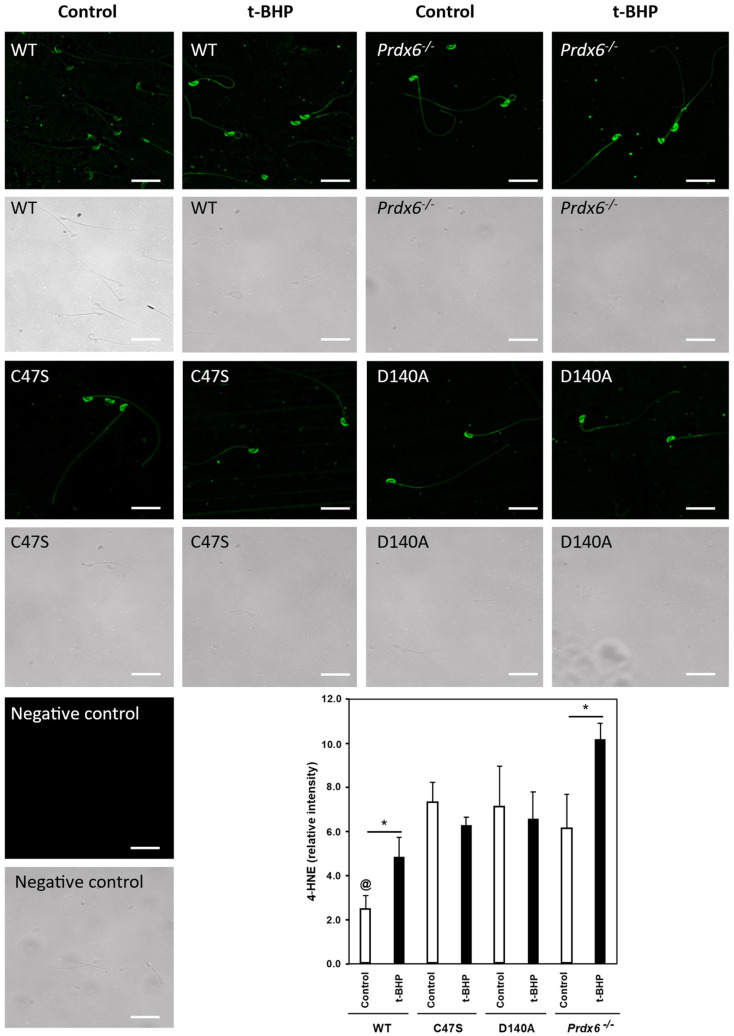
PRDX6 peroxidase or iPLA_2_ absence promotes increased levels of lipid peroxidation in the sperm plasma membrane. ImageJ was used to measure the average RFI of 4-HNE signal for >200 non-permeabilized sperm per sample. ^@^ means lower than all others. * means significant differences in the same strain. Two-way ANOVA and Bonferroni post hoc tests; *p* < 0.05 (*n* = 4 males for each group). White bars = 25 μm.

Because high levels of lipid peroxidation in the sperm plasma membrane is associated with the impairment of sperm function (sperm motility, capacitation and fertilization), we determined 4-HNE levels by immunocytochemistry in non-permeabilized spermatozoa. We found that the control wild-type spermatozoa had significantly lower 4-HNE levels compared to the spermatozoa from the control mutant strains (Figure 3). The 4-HNE levels in spermatozoa from the treated wild-type males were similar compared to the C47S and D140A control and treated males and the control *Prdx6^−/−^* males. Spermatozoa from *Prdx6^−/−^* males treated with t-BHP had the highest levels of 4-HNE among all the groups.

### 3.4. The Absence of PRDX6 Peroxidase and iPLA_2_ Activities Increased Tyrosine Nitration in Spermatozoa

**Figure 4:**
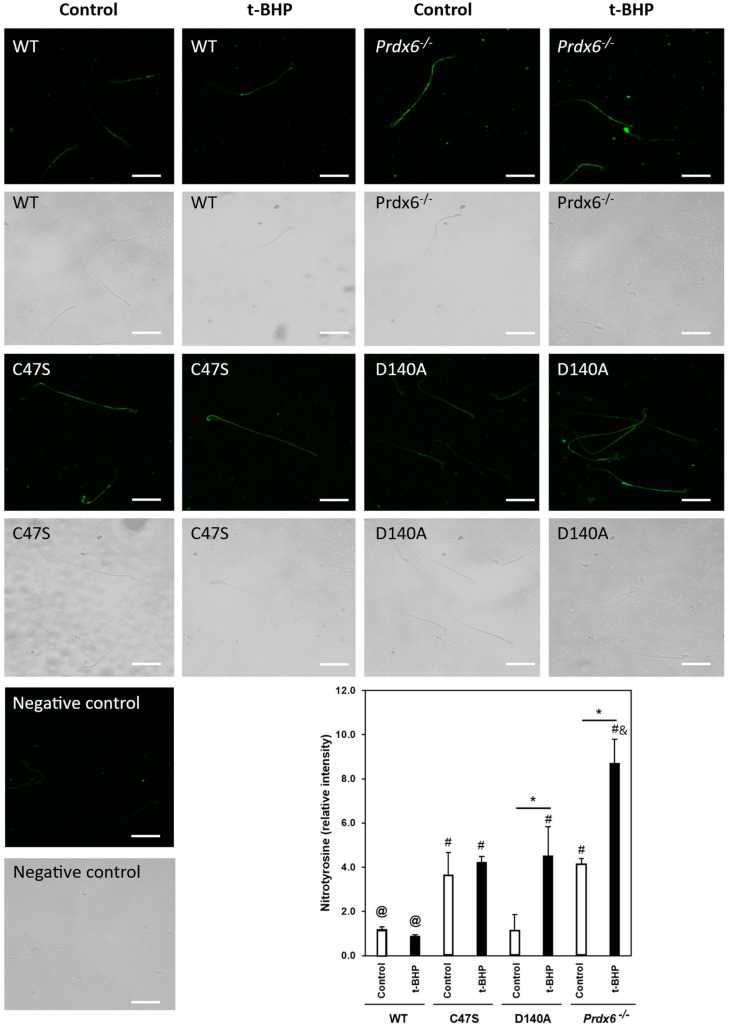
Increased protein tyrosine nitration is observed in non-permeabilized C47S and D140A spermatozoa in response to oxidative stress. ImageJ was used to measure the average RFI of nitrotyrosine signal for >200 non-permeabilized sperm per sample. ^@,&^ mean the lowest and highest values. * means significant differences in the same strain. ^#^ means higher than control wild-type (WT). Two-way ANOVA and Bonferroni post hoc tests; *p* < 0.05 (*n* = 4 males for each group). White bars = 25 μm.

Next, we wanted to know whether the levels of tyrosine nitration in spermatozoa were elevated due to the absence of PRDX6 peroxidase or iPLA_2_ activities. The immunocytochemistry studies revealed that non-permeabilized spermatozoa from the C47S control males had significantly higher tyrosine-nitration levels compared to control wild-type spermatozoa (Figure 4). Moreover, these high values of tyrosine nitration found in C47S control spermatozoa were similar to those observed in spermatozoa from control *Prdx6^−/−^* males. Interestingly, control D140A males had similar sperm tyrosine-nitration levels compared to wild-type males. The t-BHP treatment significantly increased tyrosine nitration in the D140A spermatozoa to levels that were comparable to those found in spermatozoa from C47S and *Prxd6^−/−^* males. The treated *Prdx6^−/−^* males had the highest tyrosine-nitration levels of all the groups.

### 3.5. Sperm DNA Oxidation Is Increased Due to the Absence of PRDX6 Peroxidase and iPLA_2_ Activities

**Figure 5:**
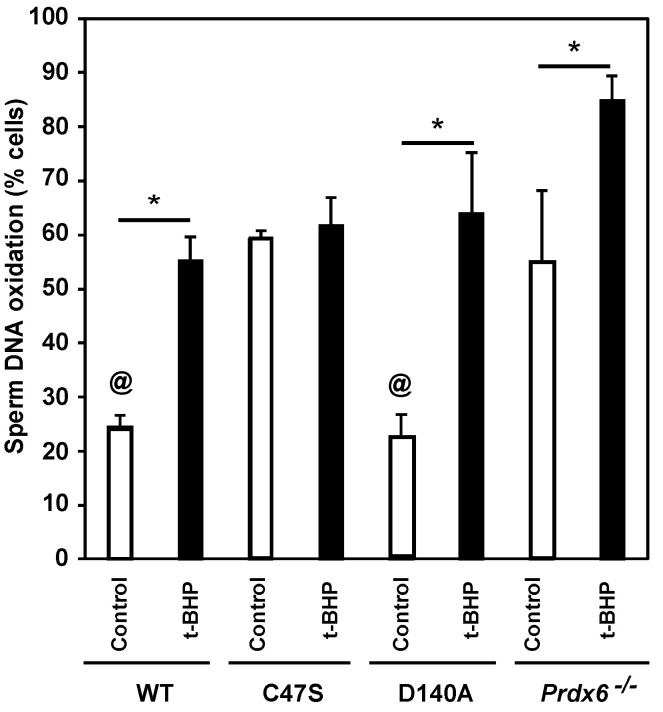
PRDX6 peroxidase or iPLA_2_ absence leads to increased sperm-DNA-oxidation levels. Sperm DNA oxidation values are presented as percentages of spermatozoa with a strong 8-OHdG signal. ^@^ means lowest among all others. * means significant differences in the same strain. Two-way ANOVA and Bonferroni post hoc tests; *p* < 0.05 (*n* = 3 males for each group).

The absence of PRDX6 is associated with increased levels of sperm DNA oxidation in mice [34,47]. Thus, we assessed the impact of the lack of PRDX6 peroxidase or iPLA_2_ activities on the integrity of the paternal genome. We observed that control wild-type and D140A males had the lowest sperm-DNA-oxidation levels among all the groups (Figure 5). Spermatozoa from the C47S control and treated males had similarly high levels of DNA oxidation as those seen in *Prdx6^−/−^* control males. The t-BHP treatment significantly increased sperm-DNA-oxidation levels in wild-type, D140A and *Prdx6^−/−^* males.

### 3.6. PRDX6 Peroxidase and iPLA_2_ Activities Are Necessary to Maintain Sperm Motility and Capacitation

**Figure 6:**
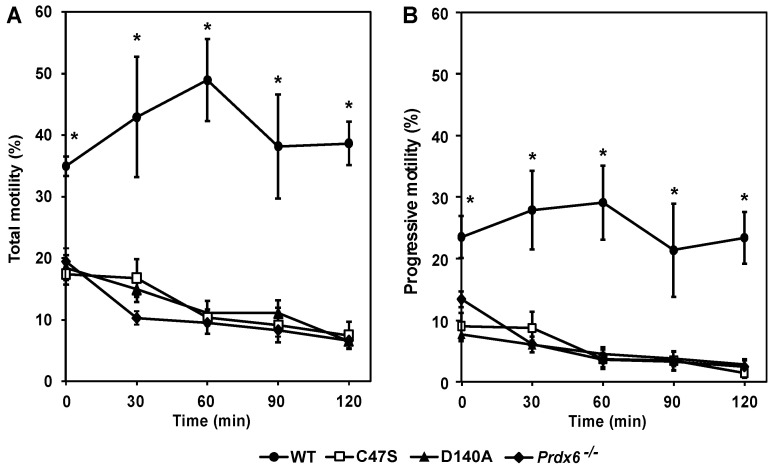
PRDX6 peroxidase or iPLA_2_ absence leads to impaired sperm motility. Parameters pertaining to total (**A**) and progressive (**B**) motility were obtained through CASA system. Thermoresistance test using PBS was used to assess sperm’s ability to cope with stress caused by lack of nutrients required for their survival. * means higher than all others at the same time. Two-way ANOVA and Bonferroni tests; *p* < 0.05 (*n* = 5 males for each strain).

Sperm motility and capacitation, which is the process required by the spermatozoon to achieve fertilizing ability, are impaired by the absence of PRDX6 in mouse spermatozoa [33,34]. The sperm total and progressive motility of C47S, D140A and *Prdx6^−/−^* males at zero-time were significantly lower compared to wild-type males (Figure 6). Sperm motility of mutant strains significantly decreased during incubation in PBS (without energetic substrates) over time and was lower than that observed in the wild-type controls. No significant differences in the total or progressive motility were observed among the mutant strains at all the time points.

**Figure 7:**
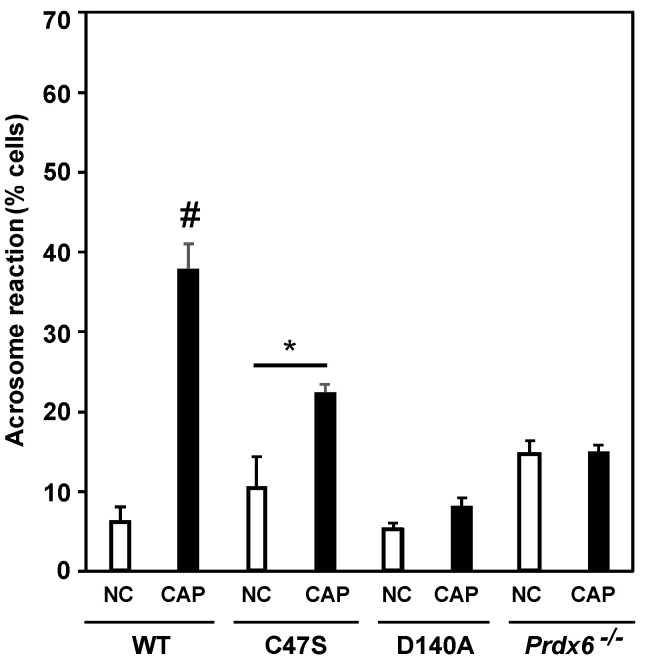
PRDX6 peroxidase or iPLA_2_ activities absence leads to impaired sperm capacitation. Capacitated (CAP) and non-capacitated (NC) spermatozoa were treated with progesterone to promote acrosome reaction. Percentages of acrosome reaction (%AR) were considered as a parameter of sperm capacitation. ^#^ means higher than all others. * means significant difference in the same strain. Two-way ANOVA and Tukey tests; *p* < 0.05 (*n* = 5 males for each strain).

Spermatozoa from C47S, D140A and *Prdx6^−/−^* males failed to capacitate when incubated with BSA/bicarbonate (well-known inducers of mouse sperm capacitation), as evidenced by the low percentage of acrosome reaction (%AR) compared to wild-type males (Figure 7). Noteworthily, the C47S spermatozoa, which were incubated with the capacitation inducers, showed an increase in %AR compared to the non-capacitated C47S controls. However, this value was lower compared to that observed in the capacitated wild-type spermatozoa.

## 4. Discussion

The results presented in this study confirm the importance of PRDX6 in supporting male-mice fertility [33,34,44,47] and demonstrate, for the first time, that PRDX6 peroxidase and iPLA_2_ activities are essential to ensure male fertility and the integrity of the paternal genome. Interestingly, we found that the absence of PRDX6 peroxidase or iPLA_2_ activities promoted the same abnormal reproductive outcome (reduction in litter numbers and pups, and a high number of infertile matings) and low sperm quality (reduction in motility, high levels of lipid peroxidation, tyrosine nitration and DNA oxidation in spermatozoa, and sperm’s inability to achieve fertilizing ability), indicating that the two PRDX6 enzymatic activities are essential to ensure male-mouse fertility.

Previous studies from our laboratory have demonstrated that the absence of PRDX6 or the inactivation of PRDX6 iPLA_2_ activity with the lipid analog MJ33 [48] result in lipid peroxidation of mouse and human spermatozoa, respectively [24,34]. Indeed, all mutant groups had higher levels of 4-HNE in the sperm plasma membrane compared to the control wild-type spermatozoa (Figure 3), thus confirming the importance of PRDX6 and its iPLA_2_ activity in preventing lipid peroxidation in spermatozoa. Interestingly, the C47S spermatozoa also had higher 4-HNE levels in their plasma membrane compared to those of control wild-type mice, and similar levels to those observed when only iPLA_2_ activity (D140A strain) or the PRDX6 protein (*Prdx6^−/−^*) were absent in spermatozoa. This increase in 4-HNE levels observed in C47S spermatozoa is due to the lack of PRDX6 peroxidase activity increasing ONOO^−^, which is capable of promoting lipid peroxidation [49]. Moreover, the inhibition of PRDX6 peroxidase activity promotes toxic levels of ONOO^−^ in human spermatozoa [24]. Thus, it is possible that the absence of PRDX6 peroxidase activity leads to levels of lipid peroxidation that overburden the PRDX6 iPLA_2_ activity and thus, impair sperm function. Altogether, these results suggest that PRDX6 peroxidase and iPLA_2_ activities are essential to protect mouse spermatozoa against lipid peroxidation. Noteworthily, the t-BHP treatment promoted higher levels of 4-HNE in spermatozoa and plasma membrane of the males lacking PRDX6 peroxidase or iPLA_2_ activity or the PRDX6 protein compared to the treated wild-type males, suggesting that spermatozoa lacking PRDX6 peroxidase or iPLA_2_ activities or the PRDX6 protein are susceptible to oxidative stress and unable to maintain low levels of lipid peroxidation, which are detrimental to sperm function.

The C47S and D140A mice experienced a stronger sperm oxidative stress compared to the wild-type mice, as suggested by the higher 4-HNE levels in the spermatozoa that were observed in both treated knock-in strains compared to the treated wild-type mice (Figure 3). These elevated sperm 4-HNE levels of C47S and D140A control mice were associated with the impairment of sperm motility and capacitation (Figure 6 and Figure 7), suggesting that 4-HNE could be responsible for the impairment of the acquisition of fertilizing ability by the spermatozoon. Sperm-plasma-membrane proteins that are modified by 4-HNE could include the voltage-dependent anion-selective channel protein 2 (VDAC2) and the Na^+^/K^+^-transporting ATPase subunit alpha-4 (ATP1A4) [50,51]. VDAC2 is responsible for allowing the influx of Ca^2+^ ions into mitochondria [50] and is found within the acrosome and principal piece of mice spermatozoa. The inhibition of VDAC2 leads to the impairment of sperm capacitation [52]. ATP1A4 is found within the sperm membrane and is involved in maintaining the balance of intracellular and extracellular sperm ion concentrations to regulate sperm intracellular pH, calcium concentrations and sperm motility [51,53]. The inhibition of ATP1A4 in rat spermatozoa resulted in the reduction in pH and calcium content, which led to motility impairment [51].

Human spermatozoa treated with the NO^•^ donor, DA-NONOate, had impaired motility and showed increased levels of tyrosine-nitrated proteins, which can be found in the midpiece (where the mitochondria are located) and the flagellum [16]. Moreover, *Prdx6^−/−^* spermatozoa displayed high tyrosine-nitration levels in the midpiece and flagellum [44]. Thus, glycolytic enzymes found in the fibrous sheath of the sperm flagellum and those of the citric-acid cycle found in mitochondria can be tyrosine nitrated [16]. Indeed, along with tubulin, these enzymes undergo tyrosine nitration during oxidative stress [16,54], thus causing the impairment of sperm motility observed in C47S, D140A and *Prdx6^−/−^* males. Furthermore, PRDX1, PRDX5 and PRDX6 are also localized within the Triton-insoluble fraction [36]; therefore, it is possible that these PRDX isoforms also undergo tyrosine nitration and become inactivated, thereby promoting oxidative stress in spermatozoa.

Previously, we demonstrated that the inhibition of glutathione-S-transferase pi (GSTpi), which is required for the reactivation of PRDX6 peroxidase activity [55,56], leads to increasing ONOO^−^ levels in spermatozoa, suggesting that PRDX6 peroxidase activity is the main ONOO^−^ scavenger in human spermatozoa [24]. *Prdx6^−/−^* mice have high levels of tyrosine nitration in their spermatozoa, which are associated with infertility. Their supplementation with a γ-tocopherol-enriched diet restored *Prdx6^−/−^* male fertility [44]. Since γ-tocopherol is an efficient ONOO^−^ scavenger (better than α-tocopherol) [49,57,58], these results suggest that ONOO^−^ is the primary culprit for the infertility phenotype observed in *Prdx6^−/−^* males. Here, we found that C47S control spermatozoa had higher tyrosine-nitration levels than wild-type controls (Figure 4) and were similar to those observed in *Prdx6^−/−^* control spermatozoa. Interestingly, the D140A spermatozoa that had intact PRDX6 peroxidase activity had similar tyrosine-nitration levels to those found in the control wild-type spermatozoa. Collectively, these results suggest that the absence of PRDX6 peroxidase is sufficient to promote the ONOO^−^-induced protein tyrosine nitration that leads to infertility, as observed in the C47S and *Prdx6^−/−^* males (Table 1 and Figure 1).

Despite the absence of PRDX6 peroxidase activity in C47S mice, tyrosine-nitration levels of the sperm plasma membrane did not increase in response to t-BHP treatment (Figure 4), suggesting that PRDX6 iPLA_2_ prevented 4-HNE production, which can disrupt mitochondrial activity and lead to increased generation of O_2_^•−^ [24,59]. The superoxide anion can react with NO^•^ to form ONOO^−^, which will increase the levels of tyrosine nitration. In contrast, the presence of PRDX6 peroxidase was not sufficient to prevent the t-BHP-induced increase in tyrosine-nitration levels in the plasma membrane in D140A spermatozoa (Figure 4). A possible explanation for this result is the fact that PRDX6 peroxidase has a high affinity for H_2_O_2_ [60]; therefore, during the oxidative stress generated by t-BHP treatment, the H_2_O_2_ produced by the 4-HNE-mediated impairment of mitochondrial activity would oxidize and rapidly inactivate PRDX6 peroxidase activity, resulting in ONOO^−^ accumulation and in the increase in tyrosine-nitration levels in the sperm plasma membrane of t-BHP-treated D140A mice. The inactivation of PRDX6 iPLA_2_ activity in human spermatozoa promotes an increase in 4-HNE levels [24]. Thus, the inability to repair oxidized sperm membranes due to the PRDX6 iPLA_2_ absence [42] will enable increased levels of 4-HNE and thus, disrupt mitochondrial function with the consequent increase in ROS including ONOO^−^ levels in treated D140A mice.

The immunocytochemistry studies revealed significant tyrosine-nitration levels in the sperm plasma membrane of mutant mice and treated wild-type mice (Figure 4). The matrix metallopeptidase 9 (MMP9) is localized in the acrosome region and participates in the mechanism of penetration of the zona pellucida, which is the glycoprotein that surrounds the oocyte, by the spermatozoon [61,62]. Tyrosine nitration activates MMP9, promoting astrocyte migration and the subsequent inflammation of the brain under high levels of ROS [63]. Thus, it is possible that premature activation of MMP9 by tyrosine nitration impairs the ability of the spermatozoon to penetrate the zona pellucida, thereby preventing the fertilization of the oocyte. Another potential tyrosine-nitration target in the plasma membrane is ERp57, which is a disulfide isomerase present in the plasma membrane in the equatorial region of the mouse spermatozoa [64] that is important for the interaction of the spermatozoa with the zona pellucida [65] and the fusion of the sperm membrane with the oolemma [64,66,67]. ERp57 is tyrosine nitrated in the amyotrophic lateral sclerosis (ALS) mouse model [68]; thus, the tyrosine nitration of ERp57 in spermatozoa may impair the sperm–egg interactions that are necessary for fertilization.

The absence of PRDX6 in mouse spermatozoa or the inhibition of PRDX6 iPLA_2_ in wild-type mouse or human spermatozoa lead to impairment of sperm capacitation [34,69]. Under capacitating conditions, spermatozoa from both the C47S and D140A mice displayed a lower percentage of acrosome reaction than capacitated wild-type spermatozoa (Figure 7), thus suggesting that PRDX6 peroxidase and iPLA_2_ activities are required for sperm capacitation. High levels of tyrosine nitration are associated with impaired capacitation in human spermatozoa [16]. The fact that the C47S spermatozoa failed to capacitate indicates that PRDX6 peroxidase activity is necessary to avoid high levels of tyrosine nitration in order to ensure the progression of capacitation in mouse spermatozoa. Interestingly, the C47S spermatozoa showed a small increase in %AR compared to the respective non-capacitated controls, suggesting that PRDX6 iPLA_2_ partially protects spermatozoa in the absence of the PRDX6 peroxidase activity, allowing some degree of capacitation (although these capacitation levels are lower than those found in capacitated wild-type spermatozoa). In agreement with this result, wild-type spermatozoa treated with MJ33, a PRDX6 iPLA_2_ inhibitor, failed to capacitate [34]. Moreover, phosphorylation of PKA substrates and of tyrosine residues, which are both essential phosphorylation events needed for sperm capacitation, were impaired in MJ33-treated human spermatozoa [69]. D-penicillamine, a ROS scavenger, was unable to prevent the disruption of capacitation-associated phosphorylation pathways in MJ33-treated wild-type spermatozoa [69]. Altogether, these results reinforce the need for PRDX6 iPLA_2_ activity to ensure sperm capacitation.

The absence of either PRDX6 peroxidase or iPLA_2_ resulted in impaired epididymal sperm maturation as evidenced by the higher percentages of spermatozoa with CDR among the C47S, D140A, and *Prdx6^−/−^* mice compared to the treated wild-type mice, and these values were exacerbated by the t-BHP treatment (Figure 2). The fact that the C47S and D140A spermatozoa showed high levels of CDR indicates that the function of the epididymis was impaired in these mutant strains and was severely affected by the t-BHP treatment. Previous research has demonstrated that the t-BHP treatment promoted elevated hyperoxidized PRDX6 levels in the rat cauda epididymis [70], which are associated with increased lipid peroxidation of epididymal epithelium and impaired epididymal function [70,71,72]. Considering that the absence of either PRDX6 peroxidase or iPLA_2_ increased lipid peroxidation levels in spermatozoa (Figure 3), it is plausible that the lack of enzymatic activities and t-BHP treatment would also promote lipid peroxidation of epididymal epithelium in mice.

The t-BHP treatment promotes increased levels of 4-HNE in the rat epididymis [71]. This increased lipid peroxidation in the epididymis results in abnormal sperm epididymal maturation, producing spermatozoa with reduced motility and high levels of DNA oxidation [70,71,73]. The increased percentages of spermatozoa carrying cytoplasmic droplets, which is a marker of epididymal maturation, indicates that the epididymal epithelium is impaired [74]; therefore, the lack of PRDX6 peroxidase or iPLA_2_ activities may promote significant levels of lipid peroxidation that will impair the epididymal epithelium, thus impairing sperm cytoplasmic absorption. Additionally, it is plausible that the lipid peroxidation of epididymal epithelium promotes the formation of abnormal epididymosomes that fail to interact with the spermatozoa present in the epididymis lumen, thus rendering them unable to transfer antioxidant enzymes to spermatozoa, resulting in the sperm’s inability to acquire the antioxidant protection that can mitigate oxidative stress [70,75].

PRDX6 peroxidase and iPLA_2_ prevent ROS-induced oxidation of DNA bases, as evidenced by the higher sperm-DNA-oxidation levels of treated C47S and D140A mice compared to the treated wild-type mice (Figure 5). These results are in agreement with the findings in human spermatozoa showing increased levels of sperm DNA oxidation when PRDX6 peroxidase or iPLA_2_ activities were inhibited [24]. Altogether, these findings reinforce the primary role of PRDX6 as a protector of the paternal genome [24]. Interestingly, D140A spermatozoa controls, which lacked PRDX6 iPLA_2_ activity, had low sperm-DNA-oxidation levels, similar to those of the untreated wild-type controls, suggesting that the PRDX6 peroxidase activity is important to maintain sperm DNA integrity. This finding is different from what we observed in humans, where PRDX6 IPLA_2_ is better at preventing sperm DNA oxidation than the peroxidase activity [24]. Thus, these results reinforce the species-specific differences in the protection of the paternal genome. Noteworthy, for all the groups, except for the control D140A mice, the trend of increase in sperm-DNA-oxidation levels was similar to that of tyrosine-nitration levels in spermatozoa (Figure 4), suggesting that ONOO^−^ may be the primary ROS responsible for sperm DNA oxidation in mouse spermatozoa. This statement is further supported by our findings that γ-tocopherol, an efficient ONOO^−^ scavenger, prevented sperm DNA oxidation and restored fertility in *Prdx6^−/−^* males [76]. The high levels of lipid peroxidation and sperm DNA oxidation observed in D140A t-BHP-treated males (Figure 3 and Figure 5) suggest that 4-HNE is also a culprit for the low quality of sperm DNA observed in these mutant strains and emphasizes what we observed in humans, i.e., that the inhibition of PRDX6 iPLA_2_ with MJ33 promotes high levels of 4-HNE and sperm DNA oxidation.

## 5. Conclusions

Sperm abnormalities recorded in this study, namely the elevated sperm 4-HNE, tyrosine nitration, DNA oxidation levels, the impaired removal of sperm cytoplasm, and the impaired sperm motility and capacitation, led to subfertility observed in both C47S and D140A males that was comparable to that observed in *Prdx6^−/−^* males. Thus, we demonstrated the essential role of the two PRDX6 enzymatic activities (peroxidase and iPLA_2_) to support fertility in male mice.

## Figures and Tables

**Table 1:** PRDX6 peroxidase or iPLA_2_ absence leads to infertile matings.

	Number of Infertile Matings	
Strain	Control	t-BHP
Wildtype	0 ^&^	5
C47S	5	10
D140A	5	10
*Prdx6^−/−^*	6	11

Males from each strain were treated with saline (control, *n* = 5) or 60 μM t-BHP/100 g of body weight (*n* = 5 males per group) for nine days, then bred with age-matched wildtype female mice thrice. The number of infertile matings was recorded post-gestation. *n* = 15 total matings for each group. ^&^ means lower than all others.

## Data Availability

Data is contained within the article.
